# Transplant Trial Watch

**DOI:** 10.3389/ti.2022.10970

**Published:** 2022-12-01

**Authors:** Simon R. Knight

**Affiliations:** ^1^ Oxford Transplant Centre, Churchill Hospital, Oxford, United Kingdom; ^2^ Centre for Evidence in Transplantation, Nuffield Department of Surgical Sciences, University of Oxford, Oxford, United Kingdom

**Keywords:** donor management, liver transplantation outcome, patient preferences, kidney transplantation, randomised controlled trial

To keep the transplantation community informed about recently published level 1 evidence in organ transplantation ESOT and the Centre for Evidence in Transplantation have developed the Transplant Trial Watch. The Transplant Trial Watch is a monthly overview of 10 new randomised controlled trials (RCTs) and systematic reviews. This page of Transplant International offers commentaries on methodological issues and clinical implications on two articles of particular interest from the CET Transplant Trial Watch monthly selection. For all high quality evidence in solid organ transplantation, visit the Transplant Library: www.transplantlibrary.com.

**Randomised Controlled Trial 1**

Donor Simvastatin Treatment is Safe and Might Improve Outcomes After Liver Transplantation: A Randomized Clinical Trial.

*by Pagano, D., et al. Transplantation 2022 [record in progress].*

## Aims

The aim of this study was to evaluate the safety and efficacy of administration of simvastatin to liver donors after brain death (DBDs) on outcomes following liver transplantation.

## Interventions

Liver allograft DBDs were randomised to receive either 80 mg of simvastatin or placebo.

## Participants

58 liver transplant recipients (>18 years) with DBDs over 18 years of age.

## Outcomes

The primary outcome was patient survival and graft survival posttransplantation. The secondary outcomes were severe complications.

## Follow-Up

180 days post-transplant.

## CET Conclusion

This small single-centre study investigated the administration of a single dose of simvastatin intraoperatively in brain-dead donors on outcomes following liver transplantation. Although recruitment stopped prematurely due to the pandemic, the study demonstrated superior graft survival in the study group with some mechanistic evidence of changes in inflammatory gene expression. The study is well designed, with use of double-blinding, placebo control and intent-to-treat analysis. In reality, it is underpowered with a significant risk of type 1 error due to the low event rate in the primary endpoint. As with any donor intervention study, it would be important to understand the impact of the intervention on all retrieved organs, not just the liver, and no reference is made to this. Nonetheless, the findings certainly warrant further investigation and the large ongoing SIGNET study in the UK should provide more insight.

## Jadad Score

5.

## Data Analysis

Modified intention-to-treat analysis.

## Allocation Concealment

Yes.

## Trial Registration

ISRCTN27083228.

## Funding Source

Non-industry funded.

**Randomised Controlled Trial 2**

Patient Preferences for Waiting Time and Kidney Quality.

*by Mehrotra, S., et al. Clinical Journal of The American Society of Nephrology: CJASN 2022 [published ahead of print].*

## Aims

This study aimed to investigate patient preferences when presented with choices between a lower-quality kidney offered today or a higher-quality kidney offered in the future.

## Interventions

Each participant was randomised to receive one of 24 sets of questions, with each set including six questions.

## Participants

605 patients who were waiting for or had received a kidney transplant.

## Outcomes

To quantify patients’ assessment of the trade-off between kidney quality and waiting time.

## Follow-Up

Not applicable.

## CET Conclusion

This is a very interesting study from the US that posed kidney transplant offer scenarios to 605 wait-list patients. Respondents adapted their assessment of a kidney offer today in light of the potential offer that may be received in later months or years. As potential waiting time for a second offer increased, the relative importance of the graft survival for the offer on the table decreased. The average respondent was willing to forgo 4–5 years of normal transplant function to prevent waiting an additional 2 years. Younger patients, and pre-dialysis patients were prepared to wait for later, better kidney offers with longer predicted graft survival. The study was conducted in the United States and it is possible that the discard rate is higher than other countries; the authors compare to France where more marginal kidneys are used for transplantation. The implication is that the results are not necessarily translatable to countries outside of the United States. Given the variability of patient preferences, it is worth having an individualised approach to kidney offer assessment adapted to each patients’ priorities. A key limitation of the study is that future kidney offers were described in terms of certainty to avoid heuristics. It is possible therefore that in real world situations patients may be even more likely to accept a marginal offer, as any future offer is not guaranteed to give better graft survival.

## Funding Source

Non-industry funded.

## Clinical Impact Summary

Decline rates for kidneys offered for transplantation vary widely between countries, transplant centres and clinicians—a reflection of uncertainty as to quality and likely outcome. These disparities usually arise from a genuine desire to do the best thing for our patients, but patients are rarely involved in depth in these decisions and it is likely that their priorities sometimes differ from those of the clinicians treating them.

In a recent paper from the US, Mehrotra et al. use a discrete choice experiment to explore patient preferences over organ offers and the impact of age, demographics and dialysis status on these preferences (1). They presented 605 patients with putative organ offers, with information about likely graft survival time and subsequent waiting list time if they were to decline the offer. They found that the average patient would accept a kidney with predicted graft survival of 6.5 years to avoid 2 years of additional waiting time for a better quality kidney with 11 years of predicted graft survival. However, younger patients and those still pre-dialysis were more likely to prefer to wait for a better kidney, and older, black or less educated patients were less willing to wait longer for a better-quality kidney.

These findings suggest that in many cases, patients would much rather go ahead with a transplant now than wait longer for something slightly better. Of course, the real world is not quite as black and white as this—there is no guarantee that a subsequent kidney offer would be better than the one currently presented. Predictions of graft survival and waiting time are not exact—the current paper uses predicted survival based upon KDPI, which has a c-statistic of 0.62 indicating only moderate predictive ability for graft survival (2). The authors recognise some limitations—particularly that patients making hypothetical decisions may act differently to a real decision, and that some included patients were post-transplant and may feel differently about risk compared to their own pre-transplant status.

If nothing else, this study demonstrates the importance of involving patients in the organ decision process. For example, the authors advocate recording patients’ risk preferences on the wait list so that these can be taken into account either during allocation or upon consideration of an offer. For this to work in real clinical practice, we need improved predictive models for transplant and wait-list outcomes, and tools to present these in a patient-friendly manner.
